# Salivary and Serum Interleukin-6: A Credible Marker for Predicting Oral Leukoplakia and Oral Squamous Cell Carcinoma by Enzyme-Linked Immunosorbent Assay (ELISA)

**DOI:** 10.7759/cureus.59113

**Published:** 2024-04-26

**Authors:** Mary Oshin, Pavan G Kulkarni, Shyam Prasad Reddy D, G Deepthi, Keerthi Sai S, Jishnu K S

**Affiliations:** 1 Department of Oral Pathology, Kamineni Institute of Dental Sciences, Narketpally, IND; 2 Department of Computing Technologies, Sri Ramaswamy Memorial (SRM) Institute of Science and Technology, Chennai, IND

**Keywords:** enzyme-linked immunosorbent assay (elisa), oral squamous cell carcinoma, oral leukoplakia, biomarker, interleukin-6

## Abstract

Background: Oral squamous cell carcinoma (OSCC) is the most prevalent subtype of oral cancer. Detecting oral potentially malignant disorders (OPMDs) in their early stages is crucial to prevent their advancement into OSCC. One of the primary factors contributing to OSCC is tobacco use, which can lead to increased production of cytokines. Among these cytokines, interleukin-6 (IL-6), an immune molecule involved in inflammation, may serve as a valuable indicator for assessing the progression of OPMDs and OSCCs.

Aims: The aim of this study is to assess the levels of IL6 in both serum and saliva using the enzyme-linked immunosorbent assay (ELISA) technique and to determine the prognostic value of these measurements in individuals with oral leukoplakia and OSCC.

Materials and methods: The research involved 45 participants, who were categorized into three groups: OSCC (15), leukoplakia (15), and a control group consisting of healthy individuals (15). Saliva and serum samples were collected from each individual within all three groups and analyzed using the ELISA method. Subsequently, the gathered data underwent statistical analysis for evaluation.

Results: There were elevated levels of IL-6 in both saliva and serum among individuals with OSCC in comparison to those with leukoplakia and the healthy control group, and this difference was statistically significant. The analysis of ROC (Receiver Operating Characteristic) curves demonstrated that salivary IL-6 was a more effective indicator than serum IL-6 for detecting the advancement of OSCC. As the histological grade of differentiation increased in both OSCC and leukoplakia cases, there was a corresponding rise in salivary IL-6 levels.

Conclusion: Both salivary and serum IL-6 levels have the potential to serve as valuable prognostic biomarkers for oral leukoplakia and OSCC which shows possible involvement of IL-6 in the development and progression of these conditions. Salivary IL-6 is a superior prognostic marker compared to serum IL-6 due to its non-invasive nature which makes it a useful tool for mass screening.

## Introduction

Oral squamous cell carcinoma (OSCC) is an invasive type of epithelial neoplasm that can develop in various parts of the oral cavity, including the gingiva, floor of the mouth, tongue, palate, and tonsils. OSCC accounts for 84-97% of oral cancers, which ranks sixth among all types of cancer, globally. India has the largest number of oral cancer cases and one-third of the total burden of oral cancer globally [1].

The progression to oral cancer typically starts with clinically noticeable oral potentially malignant disorders (OPMDs), and these conditions result from a combination of different factors. Risk factors include tobacco smoking, betel quid chewing, alcohol consumption, demographic and socioeconomic factors, inadequate oral hygiene, occupational exposure, and issues with dental prostheses of which tobacco is the most common culprit [2-3].

Tobacco usage in the Indian population is approximately 35%. The highest incidence of OSCC is observed in individuals with a history of smokeless tobacco use (45.0%), followed by those who have used both smoking and smokeless tobacco (41.8%), accounting for a combined prevalence of 86.8%. Conversely, in females, the highest frequency of OSCC occurs among smokeless tobacco users, comprising 72.8% of cases [4].

The immune response to various risk factors can trigger the production of cytokines, both locally and systemically. These cytokines may potentially serve as diagnostic and prognostic markers in cancer. Cytokines act as molecular messengers that help coordinate the actions of immune cells. Pro-inflammatory cytokines include interferon (IFN- 𝛾), interleukin-6 (IL-6), IL-1 𝛽, and tumor necrosis factor-alpha (TNF-𝛼), while anti-inflammatory cytokines include IL-10 and IL-4 [5-6].

IL-6 is produced by various types of inflammatory cells and fibroblasts. It plays a central role in host defense mechanisms. Elevated levels of IL-6 in head and neck cancer patients may independently predict tumor recurrence, poor survival, and metastasis. IL-6 facilitates communication between endothelial cells and tumor cells through chemokines, promoting the transendothelial migration of cancer cells. IL-6 plays a role in regulating tumor angiogenesis and lymphangiogenesis, contributing to tumor progression and metastasis [7-10]. 

Cytokine levels can be measured in body fluids such as serum, saliva, urine, and cerebrospinal fluid. Salivary and serum biomarkers have the potential to serve as screening tools, not dependent on the localization of a specific lesion for diagnosis. Enzyme-linked immunosorbent assay (ELISA) is widely used for detecting antigens or antibodies in body fluids, involving the use of enzymes labeled with antigens or antibodies [11]. 

In light of this background, our current study aimed to evaluate the prognostic value of salivary and serum IL-6 in OPMDs and OSCC, correlating their levels with the degrees of dysplasia and histological differentiation, respectively, using the ELISA technique.

## Materials and methods

The study comprised a total of 45 participants, and they were categorized into three distinct groups. Group A consisted of 15 patients diagnosed with OSCC. These patients were diagnosed based on both clinical examination and histopathological analysis and were classified as well, moderately, or poorly differentiated OSCC using Broder's classification. Group B comprised 15 patients who received clinical diagnosis as leukoplakia and histopathological diagnoses indicating various grades of oral epithelial dysplasia (OED). The OED grades included mild, moderate, and severe dysplasia, which were determined following the WHO's 2005 classification criteria, taking into account the presence and severity of cellular abnormalities and architectural features. Group C consisted of 15 healthy individuals, with no history of any local and systemic diseases, who served as controls. Informed consent was obtained from all the selected individuals. Institutional ethical committee clearance from Kamineni Institute of Dental Sciences, Narketpally, Nalgonda (IEC- KIDS/IEC/2017/05) was obtained for the present study.

Inclusion criteria

Individuals in Groups A and B received clinical and histopathological diagnosis and were not currently undergoing or had previously undergone any definitive treatment, such as surgery, radiation, chemotherapy, or other supplementary therapies, for their cases of OSCC or oral leukoplakia. All participants fell within the age range of 21 to 65 years, and both males and females were included. 

Exclusion criteria

Participants with preexisting systemic conditions like diabetes, hypertension, or any other underlying illnesses that would raise levels of cytokines in saliva and serum were excluded. Pregnant or breastfeeding women, individuals with a history of local therapeutic treatments like those currently using medications like anti-histamines, antihypertensives, anti-cholinergics, antidepressants, bronchodilators, and those experiencing pathological dry mouth syndrome were also not included.

From the total of 45 subjects, both saliva and serum samples were gathered, and all 90 samples underwent IL-6 estimation using the ELISA method.

Collection of salivary samples

Before providing salivary samples, participants were encouraged to fast for a minimum of one hour. Respondents were instructed to clean their mouths with water before collecting their saliva. Participants were instructed to swallow, bend their heads forward, and spit into sterile, disposable tubes. The collected saliva was centrifuged at 3,500 rpm for 10 minutes. The supernatant was then poured into 1.5 ml Eppendorf tubes and stored at -80°C.

Collection of serum samples

Serum samples were obtained via intravenous collection into a glass container using a 5ml syringe. These samples were then allowed to naturally clot at room temperature for one hour. The clot was carefully detached from the container walls to aid in retraction. The resulting serum, which has a straw-colored appearance, was collected and subjected to a five-minute centrifugation process to facilitate erythrocyte sedimentation. Subsequently, the serum was transferred to Eppendorf tubes, and these tubes were stored at -80°C [2].

Estimation of IL-6

The estimation of IL-6 levels in both saliva and serum was conducted using the De Quanto TM Human IL-6 ELISA kit, designated as # QT 4001, following the manufacturer's instructions. The technique employed was the sandwich method and was executed as follows: A volume of 100 μl of specimen samples was added to each well of a 96-well microtiter plate coated with pre-IL-6 antibodies. Following this, 50 μl of diluted biotinylated anti-IL-6 was added to all the wells. The wells were then covered with a plastic plate cover and allowed to incubate at room temperature (between 18 and 25°C) for one hour. After incubation, the covers were removed, and the plates were thoroughly washed. To each well, 100 μl of streptavidin-HRP was added and covered again with a plastic plate cover. This mixture underwent a 30-minute incubation at 25°C before being washed. After that, 100 μl of TMB substrate solution was introduced into each well and incubated in the dark at 25°C for 15 minutes. This was followed by the addition of 50 μl of stop solution (H_2_SO_4_) to each well. The resulting product displayed a color change, and the alterations in color intensity, along with absorbance readings at wavelengths of 450 nm and 600 nm, were measured using an ELISA microplate reader (EMP Emperor Medical - M201 microplate reader).

**Figure 1:**
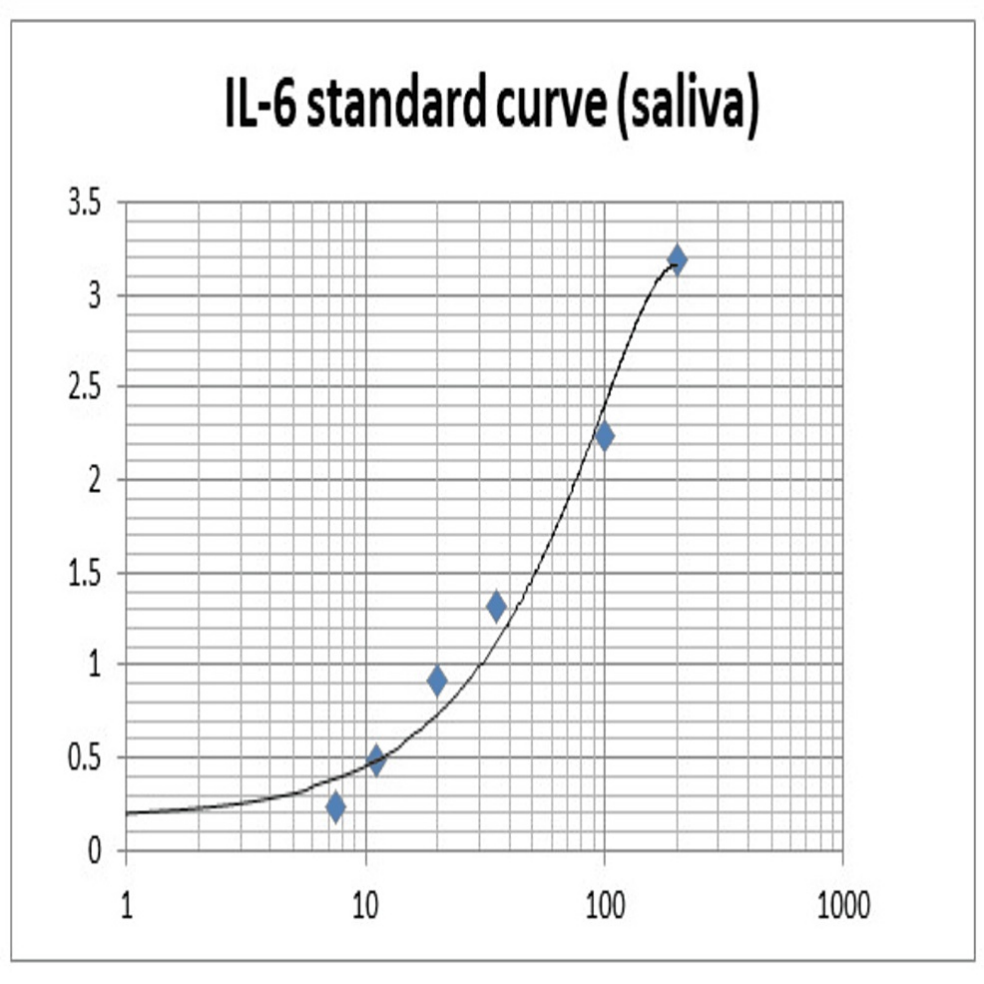
Standard curve plotted on a semi-log graph with optical density values on y-axis and concentrations (pg/ml) of interleukin-6 in saliva on x-axis pg/ml: Picograms/Milliliter Self-made image

**Figure 2:**
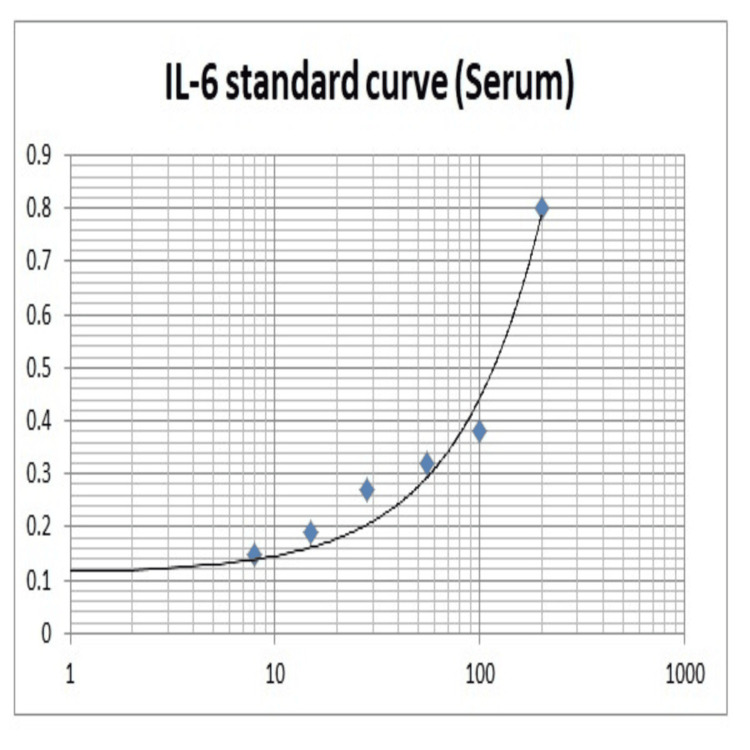
Standard curve plotted on a semi-log graph with optical density values on y-axis and concentrations (pg/ml) of interleukin-6 in serum on x-axis pg/ml: Picograms/Milliliter Self-made image

Plotting the optical density (OD) values of standards (S1-S6) against their respective concentrations resulted in the creation of a standard curve (Figures 1, 2). In order to determine the total IL-6 concentrations in picograms per milliliter (pg/ml) for both serum and saliva, the OD values of the samples taken for this investigation were then plotted on this standard curve. Statistical analysis of the generated data was performed using IBM SPSS Statistics for Windows, Version 19 (Released 2010; IBM Corp., Armonk, New York, United States).

Statistical analysis

We carried out a descriptive statistical analysis and obtained the mean, median, interquartile range, and standard deviation. We used one-way ANOVA followed by a post hoc test to evaluate group differences. The best cutoff point was determined using receiver operating characteristic (ROC) curve analysis and the maximum positive likelihood ratio (PLR), which is determined by dividing sensitivity by (1-specificity). We compared the accuracy of salivary IL-6 and serum IL-6 in determining the development from oral epithelial dysplasia to OSCC using the area under the curve (AUC). p-value, less than 0.05, was regarded as statistically significant.

## Results

The study comprised a total of 45 participants, and they were categorized into three distinct groups of OSCC, leukoplakia, and controls, each containing 15 participants. From each participant, both saliva and serum samples were collected. The 15 participants diagnosed with OSCC were within the age group of 38 to 70 years and 11 (73.3%) of them were males and 4 of them were females. Fifteen participants diagnosed with leukoplakia were within the age group of 28 to 76 years and 12 (80%) of them were males and 3 (20%) of them were females. The control group had eight (53%) males and seven (46.6%) females within the age group of 20 to 62 years (Table 1). The participants diagnosed with OSCC were histopathologically grouped into well-differentiated, moderately differentiated, and poorly differentiated squamous cell carcinoma and those diagnosed with leukoplakia were histopathologically grouped into mild, moderate, and severe dysplasia (Table 2). 

**Table 1:** Socio-demographic data of study participants OSCC: Oral squamous cell carcinoma

Socio-Demographic Data	OSCC (A)	Leukoplakia (B)	Controls (C)
Total no. of Participants	15	15	15
Total no. of samples	30	30	30
Age (range)	38- 70yrs	28-76yrs	20-62yrs
Males	11 (73.3%)	12 (80%)	08(53%)
Females	4 (26.6%)	3 (20%)	07 (46.6%)

**Table 2:** Study participants grouped based on histologic differentiation OSCC: Oral squamous cell carcinoma; NA: Not applicable

Histological Differentiation	OSCC (A)	Leukoplakia (B)	Controls (C)
Well differentiated	05	NA	NA
Moderately differentiated	06	NA	NA
Poorly differentiated	04	NA	NA
Mild dysplasia	NA	05	NA
Moderate dysplasia	NA	06	NA
Severe dysplasia	NA	04	NA

The mean serum IL-6 levels in OSCC, leukoplakia, and the control group were 61.23 pg/mL, 19.07 pg/mL, and 3.1 pg/mL, respectively, whereas the mean salivary IL-6 levels were 93.6 pg/mL, 21.04 pg/mL, and 6.91 pg/mL, respectively (Table 3). With a p-value of 0.000 (p 0.05), the rise in serum and salivary IL-6 levels in OSCC when compared to leukoplakia and the control group was very significant. Additionally, there was an increase in mean salivary and serum IL-6 levels across all grades of OSCC, from well-differentiated squamous cell carcinoma (WDSCC) to poorly differentiated squamous cell carcinoma (PDSCC), as well as across all grades of OED, from mild dysplasia to severe dysplasia (Tables 4, 5).

**Table 3:** The mean salivary and serum levels of Interleukin-6 in different groups OSCC: Oral squamous cell carcinoma; IL-6: Interleukin-6; Pg/ml: Picograms/milliliter

Groups	Mean Salivary IL-6 Level (P G/ML)	Mean Serum IL-6 Level (P G/ML)	
Group A- OSCC	93.6 ± 63.67	61.23 ± 35.11	
			Group B- LEUKOPLAKIA	21.04 ±5.56	19.07±16.81	
Group C- CONTROL	6.91 ± 5.49	3.1 ± 1.3				

**Table 4:** Comparison of salivary and serum interleukin-6 levels in different grades of oral squamous cell carcinoma (OSCC) OSCC: Oral squamous cell carcinoma; WDSCC: well-differentiated squamous cell carcinoma; MDSCC: moderately differentiated squamous cell carcinoma; PDSCC: poorly differentiated squamous cell carcinoma; IL-6: Interleukin-6; PG/ML- Picograms/Milliliter

Different Grades of OSCC	Mean Salivary IL-6 Levels (P G/ML)	Mean Serum IL-6 Levels (P G/ML)
WDSCC	43.24 ± 25.65	31.6 ± 15.5
MDSCC	81.3±36.78	64± 33.87
PDSCC	175 ± 26.18	94.12± 24.38

**Table 5:** Comparison of salivary and serum interleukin-6 levels in different grades of oral epithelial dysplasia (leukoplakia) OED: Oral epithelial dysplasia; IL-6: Interleukin-6; pg/ml: Picograms/Milliliter

Different Grades of OED (Leukoplakia)	Mean Salivary IL-6 Levels (P G/ML)	Mean Serum IL-6 Levels (P G/ML)
Mild	15.6±2.4	6.4 ±2.06
Moderate	21.4± 2.7	17.86±6.53
Severe	27.2 ± 5.4	36.62±23.76

A cutoff value of 30 pg/mL was determined from the obtained IL-6 levels by analyzing true positive and false positive results. This cutoff value represents the threshold where there is the highest number of true positive results and the lowest number of false positive results. In this study, a cutoff value of 30 pg/ml was identified for both saliva and serum. Sensitivity and specificity were calculated at this threshold and demonstrated 86% sensitivity and 93% specificity in distinguishing between OPMDs and OSCC, while serum IL-6 showed 80% sensitivity and 86% specificity for the same purpose.

**Figure 3:**
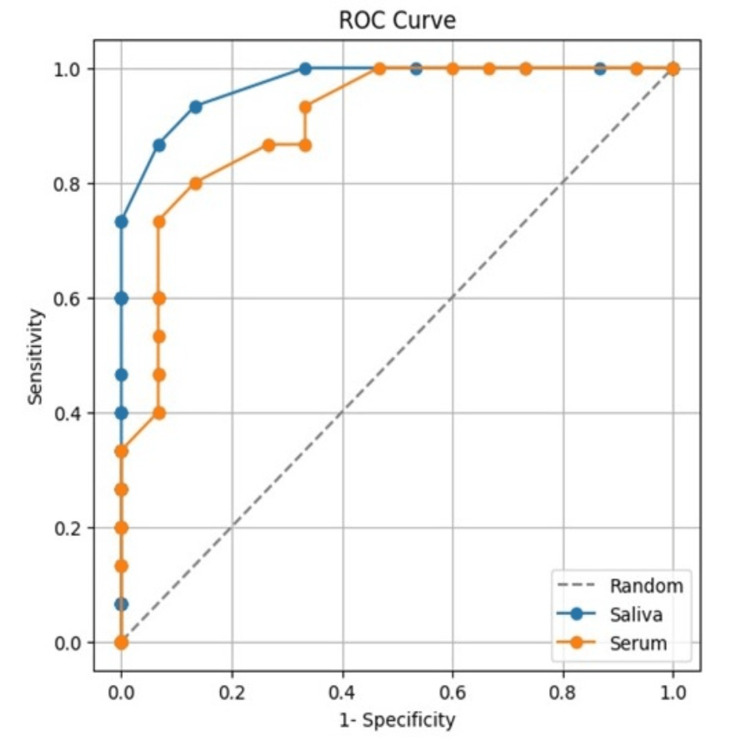
Sensitivity and specificity of interleukin-6 assessed by ROC curve analysis. ROC curve analysis of IL-6 marker in saliva and serum to compare the accuracy of salivary IL-6 and serum IL-6 in detecting the progression of oral epithelial dysplasia to oral squamous cell carcinoma ROC curve: Receiver operating characteristic curve Self-made image

The area under the ROC curve for salivary IL-6 was greater than that for serum IL-6, indicating that salivary IL-6 is more accurate than serum IL-6 in detecting the progression from OED to OSCC (Figure 3).

## Discussion

OSCC is characterized by the presence of tumor epithelium and the connective tissue stroma, collectively forming the tumor microenvironment (TME). This TME is made up of a variety of components, including inflammatory cells, extracellular matrix, and mesenchymal cells. Communication between tumor cells and stromal elements supports tumor genesis, growth, invasion, and metastasis through the TME. Inflammatory cytokines like TNF-α and IL-6 serve as a conduit for this communication and contribute to the recruitment of more inflammatory cells into the TME, increasing the growth and survival of genetically modified tumor cells [12]. 

The intrinsic actions of IL-6 on tumor cells have an impact on the development of cancer by affecting cell survival, proliferation, and metastatic dissemination. Additionally, by promoting angiogenesis and helping the tumor avoid immune monitoring, IL-6 exerts an extrinsic effect on other cells inside the TME, resulting in a pro-tumor environment. IL-6 has been said to have anti-inflammatory properties, but it plays a crucial role in increasing inflammation and immunological responses during tumor development [13-14]. 

The OSCC group in this study had considerably greater mean salivary IL-6 concentrations than the Leukoplakia and control groups, with a p-value of 0.000 (p 0.05). These findings align with previous studies by Katakura et al. [15], Jamee et al. [16], Brailo et al. [17], and Dineshkumar et al. [2].

Similar to this, OSCC had significantly higher mean blood IL-6 concentrations than the Leukoplakia and control groups, with a p-value of 0.000 (p 0.05). These results are consistent with studies conducted by Hamad et al. [18] and Dineshkumar et al. [2]. No significant differences in salivary and serum IL-6 concentrations were observed between patients with leukoplakia and the control group, which is consistent with the study by Brailo et al. [17].

Additionally, this study assessed the levels of salivary and serum IL-6 in various OPMD dysplasia grades and various histological grades of OSCC. The findings demonstrated a rise in mean salivary and serum IL-6 levels across different stages of OSCC, from WDSCC to PDSCC, as well as across different grades of OED, from mild dysplasia to severe dysplasia.

The mean salivary IL-6 levels in mild dysplasia were 15.66 pg/ml, in moderate dysplasia were 21.433 pg/ml, and in severe dysplasia were 27.20 pg/ml. In serum, the mean IL-6 levels in mild dysplasia were 6.48 pg/ml, in moderate dysplasia were 17.86 pg/ml, and in severe dysplasia were 36.62 pg/ml. These elevated levels of salivary and serum IL-6 in OED indicate the involvement of IL-6 in the progression to more severe forms of OED. Additionally, there was an increase in mean salivary IL-6 levels across different grades of OSCC, with mean values of 43.24 pg/ml for WDSCC, 81.30 pg/ml for moderately differentiated squamous cell carcinoma (MDSCC), and 175.0 pg/ml for PDSCC. Mean serum IL-6 levels also showed a similar trend, with values of 31.60 pg/ml for WDSCC, 64.00 pg/ml for MDSCC, and 94.12 pg/ml for PDSCC, suggesting a role for IL-6 in the progression and invasion of tumor disease. These findings are in agreement with the study by Dineshkumar et al. [2].

According to this study, saliva demonstrates higher sensitivity and specificity in detecting the progression of OSCC. This may be attributed to the increased localized production of IL-6 around tumor cells, contributing to elevated IL-6 levels in saliva. Saliva collection is a non-invasive procedure and can effectively aid in the early detection of OPMDS and prevent its progression to OSCC [2].

The following mechanism can account for the increased serum and salivary levels of IL-6 in OED and OSCC: Chemokine IL-6 plays a crucial role in the body's defense processes. T-regulatory cells and myeloid-derived suppressor cells are both simultaneously upregulated by IL-6, which boosts IL-6 production resulting in a diminished immune response to the tumor. The lesion develops an immunosuppressed TME despite the input of immune cells [19].

**Figure 4:**
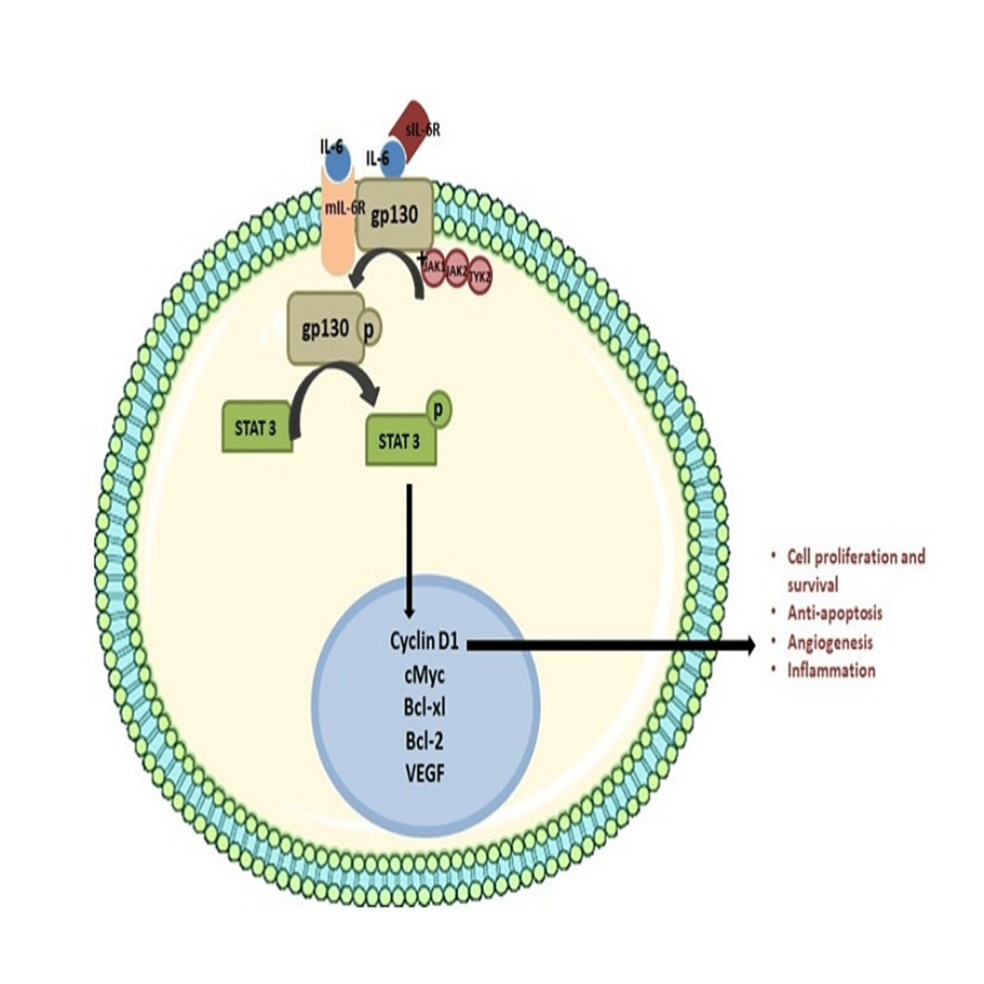
Interleukin-6 plays an important role in growth and progression into cancer. The picture illustrates the contribution of interleukin-6 through a classical and trans-signaling pathway in the progression of leukoplakia and cancer Self-made image

IL-6 normally binds to the IL-6 receptor subunit (IL-6R), which starts the IL-6 signaling process. Then, this IL-6/IL-6R complex attaches to the glycoprotein 130 (gp130), a signaling subunit. The types of cells that can react to IL-6 are largely restricted to hepatocytes, lymphocytes, and macrophages due to IL-6R expression. To activate signaling, IL-6 also uses a trans-signaling method that depends on the soluble IL-6R (sIL-6R) binding to gp130. Multiple inflammatory conditions including malignancies have markedly elevated sIL-6R levels. Gp130 is connected to kinases including JAK1, JAK2, and tyrosine kinase 2 (TYK2), which are reciprocally transphosphorylated to cause Gp130 to become phosphorylated. Phosphorylated Gp130 interacts with STAT3, which phosphorylates STAT3 and activates it. Activated STAT3 functions as a transcription factor targeting regulatory sequences of genes encoding pro-proliferation, pro-survival, and pro-angiogenic proteins in the nucleus thus aiding in the progression of OPMDs into cancer (Figure 4) [20-23].

The increased IL-6 levels in the TME are a reflection of the substantial correlation between inflammation and malignancy. By controlling a number of cancer-related processes, including apoptosis, proliferation, angiogenesis, invasiveness, metastasis, and metabolism, IL-6 encourages the growth of tumors. Additionally, IL-6 promotes repair processes and inhibits cell death signals, protecting cancer cells from oxidative stress, apoptosis, and DNA damage brought on by therapy [24-25].

The study has various limitations as it includes only pre-treated newly diagnosed cases of oral leukoplakia and OSCC and the correlation between salivary and serum IL-6 levels following the treatment was not done. Further studies correlating with follow-up of these cases have to be done, to analyze the effect of treatment modalities. The study is also limited as it includes only leukoplakia as a premalignant lesion for comparison with OSCC and other premalignant disorders like erythroplakia, lichen planus, or oral submucous fibrosis are not included in the study. Further studies with more sample size correlating with clinical staging have to be done for better results.

## Conclusions

In conclusion, this study underscores the potential significance of IL-6 as a valuable biomarker in oral leukoplakia and OSCC. The findings indicate that IL-6 may play a pivotal part in the progression of oral leukoplakia and OSCC, positioning it as a promising prognostic marker. The non-invasive nature of salivary IL-6 measurement makes it a superior tool, offering great utility in mass screening efforts, especially within populations with a heightened prevalence of potentially malignant oral disorders linked to tobacco use.
